# Nitrous Oxide Gas as an Anæsthetic

**Published:** 1868-11

**Authors:** 


### ARTICLE V.

### *Nitrous Oxide Gas as an Anæsthetic.*

[We have received the following paper from a dentist of long experience and large practice, and give it as the other side of the question. Eds.]

Dear Doctor:—After an experience of over two years with this gas, I feel impelled to give to the public the results of my observation. I am often asked the question, "does it relieve pain?" I unhesitatively and emphatically answer, "no." Then how is it that operations are performed without the knowledge of the patient. The fact is that the memory is in fault. Most operators desire the friends of patient to remain in another room, and some make it a rule that an apartment must always intervene between them, so that any screams or struggles may not be over-heard. It is not invariable that the patient struggles, and some become so dead,—yes that is the word—that there is a total loss of all voluntary motion, and hence they are quiet.

Secondly. What is the effect of the gas? Now the common statement that because it is composed of the same elements as the atmosphere, and must therefore be harmless, is ridiculous nonsense. I ask what is Nitric Acid, and other deadly poisons that could be mentioned?

The patient breathes the gas for awhile perhaps quietly, but as the blood becomes charged with carbonic acid, the demand for air is urgent, the breathing is spasmodic, the eyes protrude or roll fearfully in their sockets, the face is livid, and stertorous breathing commences. The operator, to allay the fears of friends who may be present, calls this "snoring." But it is nothing less than stertorous breathing, the same that acompanies apoplexy, and the patient is in a state of asphyxia as much so, as if he had been hanged by the neck until this state of things was produced. This is death, the insensibility produced is *death.*

I am aware that this is strong language, but it is true. Who can doubt it that has had any experience in the use of this gas, who does not know that by persisting in its exhibition only for a very few minutes after this stertorous breathing begins, actual death is the invariable, the inevitable result. As it is by a timely removal of the poisonous agents, and the admission of fresh air the patient in most cases recovers.

Now it is not for me, or for any one to say how often this state of things may be produced with impunity. In many cases no bad result is apparent. I say apparent because it may prove the remote cause of disease. These bodies of ours are of such delicate construction, that they cannot be tampered with carelessly, or the wheels of life arrested in their motion so suddenly and violently without jar or injury.

So far I have not spoken of the stimulating effect of this gas. This is transient and of minor importance, never producing symptoms such as we have described.

I could strengthen these statements by a description of many individual cases in which most unpleasant results have followed the administration of this gas, but I forbear.

It might be said that my patients are rebellious and perverse that they do not take to choking kindly, so no more at present.

Yours Truly, H.

